# Mapping family involvement in music therapy for children and adolescents with cancer: a scoping review

**DOI:** 10.1080/16549716.2026.2674493

**Published:** 2026-07-09

**Authors:** Constance Boyde, Lara-Luna Ehrenschneider, Jana Brückner, Joy-Mariella Donisch, Mara Siebenmorgen, Katharina Thomas, Christina Hunger-Schoppe

**Affiliations:** a Faculty of Health, Department of Psychology and Psychotherapy, Chair of Clinical Psychology and Psychotherapy III, Witten/Herdecke University, Witten, Germany; b Department of Paediatrics and Adolescent Medicine, Gemeinschaftskrankenhaus Herdecke, Herdecke, Germany; c Interprofessional Graduate School of Integrative Medicine and Health Sciences (IGIM), Witten/Herdecke University, Witten, Germany

**Keywords:** Music therapy, family, paediatric oncology, global child health, psychosocial care

## Abstract

Children and adolescents undergoing cancer treatment, along with their families, experience psychological and emotional distress. Music therapy is increasingly acknowledged as an intervention that may foster emotion regulation, coping, and communication; however, family involvement remains insufficiently delineated. This scoping review maps evidence on family involvement in music therapy for paediatric oncology, characterises the forms and intensity of participation, summarises intervention features and reported outcomes, and identifies limitations and research gaps. Following PRISMA-ScR guidelines, a systematic search was conducted in PSYNDEX, PubMed, The Cochrane Library, and Embase (2010–2025). Studies were eligible if they examined music therapy interventions or structured music-therapy-based interventions in paediatric oncology and involved family members at any level of participation. Eighteen studies met the inclusion criteria and were synthesised descriptively. Across the studies, reported outcomes included reductions in anxiety, stress, and pain, as well as improvements in mood, quality of life, and family interaction. Family involvement ranged from primarily observational and reflective roles to direct participation in shared music-making and, in some cases, therapist-guided parent-led facilitation. Interaction-focused and parent-directed approaches were most often described as supporting emotional connectedness, shared coping, and family resilience. Methodological heterogeneity, small sample sizes, and inconsistent conceptualisations of family involvement limited comparability. The reviewed literature suggests that family involvement in music therapy may support psychosocial well-being and relational functioning within paediatric oncology. However, the available evidence is heterogeneous and primarily descriptive. Future research would benefit from clearer conceptualisations of family involvement and more methodologically comparable studies to clarify mechanisms of change and long-term effects.

## Paper Context

- **Main findings:** This scoping review of published studies found that involving family members in music therapy for children and adolescents with cancer is commonly linked to reduced distress and improved family interaction, although approaches and outcomes vary widely.
- **Added knowledge:** The review maps clear differences in how family involvement is delivered, from observing to actively co-creating, and shows that inconsistent descriptions and heterogeneous study designs limit comparability across studies.
- **Global health impact for policy and action:** Integrating family-oriented music therapy into routine paediatric oncology care pathways and strengthening sustainable service provision can support equitable access, while future research should use clearer intervention descriptions and more comparable outcome measures to inform implementation.

## Background

In a broader global health context, more than 275,000 children and adolescents aged 0–19 years are diagnosed with cancer worldwide each year [1]. Marked inequities persist across health systems: in high-income countries more than 80% of children with cancer are cured, whereas in many low- and middle-income countries survival remains below 30% [2]. Beyond the physical consequences of treatment, the diagnosis imposes substantial psychosocial strain on the entire family system. Parents and siblings frequently experience anxiety, helplessness, disrupted routines, and social isolation, which may affect family communication, coping, and adaptation processes [3,4].

Music therapy has become an established component of psychosocial care in paediatric oncology and is recognised in national clinical guidelines [5,6]. It is understood here as the creative and professionally informed use of music within a therapeutic relationship [7], a nonverbal and emotionally intuitive form of expression and interaction through sound and improvisation [8]. This experiential mode can provide children with a safe space to explore emotions related to illness while also enabling family members to participate in meaningful, shared experiences. In situations of severe illness, family participation may be particularly relevant because parents and other family members often provide immediate emotional and practical support and play an important role in adaptation throughout the illness trajectory [3,4,9]. From a family-centred perspective, involving family members in music therapy may therefore support not only the child’s experience of care, but also shared coping, communication, and family connectedness [9,10].

Recent reviews in adjacent paediatric medical fields underscore the relevance of this perspective. Overå’s scoping review on music therapy in paediatric palliative care highlighted limited and conceptually heterogeneous research, the absence of a clear definition, and the importance of connectedness and quality of life [10]. Kammin et al.’s systematic review and qualitative evidence synthesis identified themes including emotional and physical reprieve, enhanced family wellbeing, and the centrality of the therapeutic relationship [9]. These reviews demonstrate that music therapy in paediatric medical contexts has already been systematically examined, including child-, family-, and stakeholder-related perspectives [9,10]. However, they focus on paediatric palliative care more broadly or on stakeholder experiences, rather than specifically examining how family involvement is conceptualised and enacted within paediatric oncology music therapy [9,10].

Recent professional recommendations emphasise the integration of family members into music therapy sessions to strengthen communication, emotional connection, and mutual support [6]. However, surveys indicate that only a minority of paediatric oncology units offer music therapy, and reimbursement within the statutory health care system is typically limited to individual sessions [11]. Despite clear evidence that parents and siblings are psychosocially affected by a child’s illness, they are seldom systematically included in music therapy or additional psychosocial care [4,12].

This imbalance between established clinical relevance and limited structural implementation highlights the need for a systematic synthesis of existing evidence on how families are involved in paediatric oncology music therapy. In the present review, family involvement is used as an umbrella term referring to the different ways family members are present, engaged, or actively participating in music therapy processes, ranging from supportive presence and observation to shared music-making and more active facilitative roles. Such an overview is essential to delineate current practices, conceptual frameworks, and methodological approaches, and to inform future research and policy aimed at strengthening family-integrated music therapy. From a global child health perspective, this question is also relevant for settings in which formal psychosocial infrastructures are limited, and families carry a substantial share of the emotional and practical burden of care [2,13].

## Objectives and research questions

The primary objective of this scoping review is to map how family involvement is conceptualised and operationalised in music therapy interventions for children and adolescents with cancer. Secondary objectives are to describe the forms, roles, and intensity of family participation; to summarise intervention characteristics and reported child-, family, and relational outcomes; and to identify methodological and conceptual gaps in the literature.

The following research questions (RQ) guide this study:

RQ1. How is family involvement conceptualised and operationalised in music therapy interventions within paediatric oncology?

RQ2. What forms, roles, and levels of family participation are described in the included studies?

RQ3. What intervention characteristics, reported outcomes, and methodological gaps are identified in the existing literature?

## Methods

### Protocol and registration

The review protocol was developed in accordance with the *Preferred Reporting Items for Systematic Reviews and Meta-Analyses extension for Scoping Reviews* (PRISMA-ScR) guidelines. The final protocol was prospectively registered with the *Open Science Framework* (OSF: https://osf.io/2t8xg/overview) prior to data extraction to ensure methodological transparency and reproducibility.

### Literature search and identification of relevant studies

#### Data sources

For the systematic search, the databases *PSYNDEX, PubMed, The Cochrane Library* and *Embase* were searched with a time limitation from 2010 to 2025 (last search 31 January 2025). The search was limited to studies published from 2010 onwards to focus the review on literature most likely to reflect contemporary patient- and family-centred paediatric care [14], current psychosocial oncology standards for children with cancer and their families [6,15], and more comparable reporting of music-based interventions [16]. While earlier studies may still be conceptually relevant, they were outside the scope of this review, which aimed to map the contemporary state of the literature. In addition, the professional associations *mdw/mt-family, PSAPOH, OEGPS, DKPM, SG, DGPs* and *DGSF* were contacted by circular letters to identify published and unpublished grey literature.

#### Inclusion criteria

Publications were included if they: 1) were written in English or German; 2) were published between 2010 and 2025; 3) reported on the design, implementation, or evaluation of studies investigating music therapy interventions; 4) focused on paediatric oncology; and 5) involved family members in the therapeutic process. For this review, eligible interventions were those described by the authors as music therapy or as structured music-therapy-based interventions in paediatric oncology. Across the included studies, interventions were predominantly delivered by trained, credentialed, or professional music therapists; family- or parent-delivered components were included when they were embedded within a music therapy framework and supported by therapist guidance, modelling, or protocolized materials.

#### Exclusion criteria

Publications not meeting these criteria were excluded. Only primary empirical studies were eligible for inclusion. Review articles (including systematic reviews and scoping reviews), conference abstracts, study registrations without an accompanying study protocol or full-text publication, and narrative experience reports were excluded from the analysis.

#### Search strategy and algorithms

The online databases were systematically searched using a pre-defined algorithm developed to address the research questions. The search terms were grouped into four main categories: 1) music therapy: ‘music therapy’ OR ‘music intervention’ OR ‘musical therapy’ OR ‘music based’, 2) oncology: ‘oncology’ OR ‘cancer*’ OR ‘cancer patients’, 3) paediatrics: ‘children’ OR ‘paediatric’ OR ‘pediatric’ OR ‘child*’, and 4) family: ‘famil*’ OR ‘parent*’ OR ‘mother’ OR ‘father’ OR ‘caregiver’. The search was conducted exactly as reported in Table 1, with the four term blocks combined stepwise using AND. All searches were performed in the Abstract field using free-text terms only; no controlled indexing vocabulary was applied. Searches were limited to studies published between 2010 and 2025 in English or German.

**Table 1.** Keyword-based literature search.

#				Results
**PSYNDEX Literature with PSYNDEX Tests, APA PsycArticles, APA PsycInfo via EBSCO Host (2010–2025, retrieved 31 January 2025)**				
1.		Abstract	‘Music therapy’ OR ‘music intervention’ OR ‘musical therapy’ OR ‘music based’	3,498
2.	AND	Abstract	oncology OR cancer* OR ‘cancer patients’	155
3.	AND	Abstract	children OR paediatric OR pediatric OR child*	33
4.	AND	Abstract	famil* OR parent* OR mother OR father OR caregiver	14
**Pubmed (2010–2025, retrieved 31 January 2025)**				
1.		Abstract	‘Music therapy’ OR ‘music intervention’ OR ‘musical therapy’ OR ‘music based’	3,523
2.	AND	Abstract	oncology OR cancer* OR ‘cancer patients’	336
3.	AND	Abstract	children OR paediatric OR pediatric OR child*	58
4.	AND	Abstract	famil* OR parent* OR mother OR father OR caregiver	30
**The Cochrane Library (2010–2025, retrieved 31 January 2025)**				
1.		Abstract	‘Music therapy’ OR ‘music intervention’ OR ‘musical therapy’ OR ‘music based’	2,491
2.	AND	Abstract	oncology OR cancer* OR ‘cancer patients’	263
3.	AND	Abstract	children OR paediatric OR pediatric OR child*	28
4.	AND	Abstract	famil* OR parent* OR mother OR father OR caregiver	14
**Embase (2010–2025, retrieved 31 January 2025)**				
1.		Abstract	‘Music therapy’ OR ‘music intervention’ OR ‘musical therapy’ OR ‘music based’	4,516
2.	AND	Abstract	oncology OR cancer* OR ‘cancer patients’	505
3.	AND	Abstract	children OR paediatric OR pediatric OR child*	82
4.	AND	Abstract	famil* OR parent* OR mother OR father OR caregiver	47

#### Search results

The database search identified 105 records in *PSYNDEX, PubMed, The Cochrane Library* and *Embase*. The enquiries to professional associations yielded 0 additional records (Table 1).

### Selection process of the studies

Title/abstract screening and full-text assessment were conducted by a single reviewer using the predefined inclusion and exclusion criteria.

#### Exclusion of duplicates

The identified studies were read and processed using the reference management software *EndNote*. Duplicates encompassed 31 studies, excluded prior to the actual screening process.

#### Abstract screening

Of the remaining 74 studies, 41 studies were excluded based on the inclusion and exclusion criteria following abstract screening. 18 studies did not address music therapy interventions or did so only partially [17–34]. 9 studies did not, or only partially, focus on paediatric oncology [35–43]. 8 articles contained economic or sociodemographic surveys [11,44–50]. 2 studies were abstracts for symposia [51,52]. 2 articles review studies on music therapy in paediatric oncology: one to develop guidelines [53], and the other to review the validity of music therapy interventions [54]. 2 studies focused on music therapy in the context of grief support [55] and music therapy outside the family system [56].

#### Full-text screening

The remaining 33 full texts were assessed for accessibility. 6 articles had to be excluded because they were not obtainable, even after contacting the authors directly.

**Figure 1.**
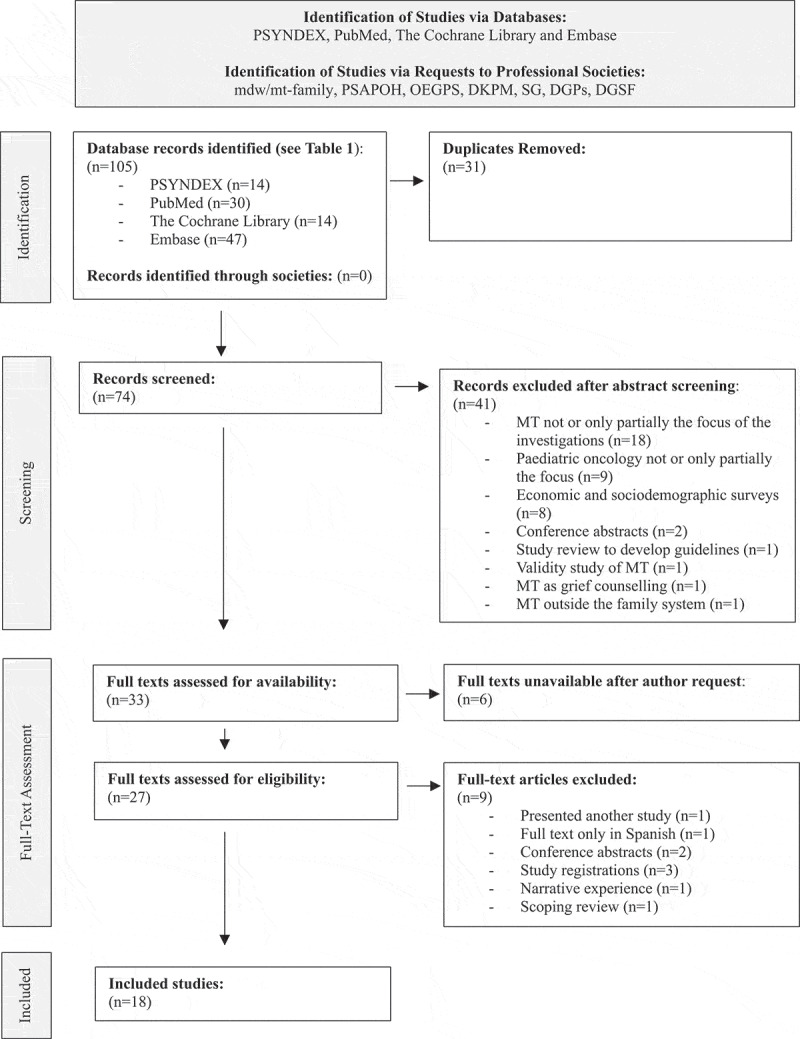
Flowchart.

Subsequently, 27 articles were examined against the inclusion criteria. Nine studies were excluded because they referred to other research [57], the full text was not available in English [58], or they were conference proceedings [59,60], study registrations [61–63], narrative experience reports [64], or an existing scoping review [65]. Ultimately, 18 studies met all inclusion criteria and were included in the data analysis (Figure 1).

### Data extraction and categorisation

The 18 datasets identified are recorded in tabular form, analysed descriptively, and categorised according to the following dimensions: *Author and Year, Country, Study Design, Objectives, Main Results*, and *Family Involvement* (Table 2); *Participants, Diagnosis, Location of Interventions, Outcome Measures*, and *Interventions* (Table 3); as well as *Implications and Recommendations for Future Research and Clinical Practice* (Table 4).

**Table 2.** Summary of included studies.

#	Author and Year	Country	Study Design	Objectives	Main Results	Family Involvement
1	Barry et al., 2010 [76]	Australia	Mixed methods	Multiple perspectives on the effect of a self-made music therapy CD (MTCD) for stress reduction during initial radiotherapy.	67% of CG reported social withdrawal as a coping strategy, but none of IG, with a near significant result (*p* = 0.076). The Kidcope tool showed no significant difference in distress scores. The MTCD creation process was enjoyed by the children. It reduced tension, improved communication and psychosocial support, and enhanced the treatment experience.	Family members were present during the MTCD process. They provided an opportunity for sharing experiences and emotional support. After the treatment, family members provided feedback. They described the interventions as helpful and enjoyable for both the child and the family members, which reduced anxiety.
2	Boyde et al., 2024 [70]	Germany	Multicentre RCT, mixed methods	Improvement of parent-child interaction, QoL, system-related functioning, psychosocial and psychosomatic distress and resources by involving family members in interaction focused Music Therapy. Examination of feasibility criteria.	Unknown (study not yet completed).	Family members are actively and equally involved in the music assessments as well as the IG MT intervention processes.
3	Fedhila et al. 2023 [73]	Tunisia	Quasi-experimental study	Assessing the impact of MT on QoL in children with cancer and its effect on cardiorespiratory rate.	Median Total QoL score increased, showing fewer symptoms of pain, nausea, anxiety and worry. A significant decrease in respiratory rate and heart rate was observed after MT. Social communication improved.	The parents were not actively involved in the MT but completed the PedsQL in 13 cases. Questionnaires asked about communication in the family system.
4	Giordano et al. 2021 [75]	Italy	Quantitative, observational, and comparative study	Comparing anxiety and sedation levels in paediatric patients undergoing invasive procedures before (T1), during (T2) and after (T3) the pandemic with and without MT.	The absence of MT due to the pandemic resulted in higher levels of pre-operative anxiety in paediatric patients undergoing invasive procedures and increased use of anxiolytic drugs for sedation. Higher levels of anxiety and stress were also found in mothers.	Mothers were interviewed about the effects of MT, and the study also found that MT has a positive effect on the mother-child dyad. The exact participation in the MT process was not described in detail.
5	Heiderscheit 2022 [77]	USA	Feasibility and acceptability study	Exploring the feasibility and acceptability of a patient–and family–directed active music-making protocol during the bone marrow transplant process.	Active music can be used to manage pain, discomfort, stress, anxiety and boredom, to promote relaxation and sleep, for enjoyment and to connect.	Study participants said they played music with multiple family members 80% of the time, including parents, siblings and grandparents. Parents/caregivers said they played 30% of the time when the patient felt weak, tired or nauseous.
6	Mahoney 2019 [78]	USA	Single case study	To describe individual support for a patient and the experiences resulting from it.	The opportunity to express creativity and connect with others made MT an important resource.	Active involvement of family members, initially reflective, then active in coping with loss and grief.
7	O’Callaghan et al. 2010 [7]	Australia	Constructivist research approach	Perspectives of paediatric cancer patients and their parents on the role of music and music therapy in children’s lives.	Music helps children with cancer and promotes their wellbeing. It can also be used in family, social and electronic contexts to encourage normal development. Music therapy is effective in reducing children’s distress and can be a source of support for families.	Parents were asked about their children’s musical interventions and benefits of their own active participation.
8	O’Callaghan et al. 2016 [79]	Australia	Meta-ethnography	Evaluation of five studies to improve the understanding of music’s broad relevance for those affected by cancer.	Music usage can remain incidental or change due to cancer. Music can support well-being or become difficult to experience. It can help or intrude, extending self-awareness and social connections, and prompting memories and legacies. Music therapists may help patients and carers recover or extend music’s effects.	Parents, family carers and bereaved relatives took part in individual studies and reported on their experiences with music in the context of cancer. Music provided emotional support, helped cope with stress, enabled expression of feelings and promoted family bonding. It could also be challenging, e.g. by bringing back painful memories. The synthesis shows that music is an important psychosocial resource for patients and their families.
9	Rodriguez-Rodriguez et al. 2023 [80]	Spain	Qualitative Participatory Action Research	To explore and transform the emotional responses of children with cancer using music therapy, and to assess how music therapy could support these children during treatment and benefit their families indirectly.	MT helped children with cancer express their emotions, leading to improved moods and emotional well-being. Sessions also encouraged social interaction and emotional release in a positive and supportive environment. Parents observed positive emotional changes in their children.	Parents are involved through observation and describe the social benefits of MT in interviews.
10	Uggla 2019 [81]	Sweden	Doctoral thesis	Evaluation of four studies about the effects of music therapy on children undergoing haematopoietic stem cell transplantation from multiple perspectives including physiological, psychological, and relational outcomes.	MT reduces physiological signs of stress, improves health-related quality of life during and after hospital treatment, enhances emotional well-being and self-expression in children, supports parental engagement and coping and increases staff awareness and compassion in the care environment.	Children could choose to involve parents or siblings, fostering emotional connection. Parents took part in interviews and completed quality of life questionnaires. The sessions offered relief, bonding and emotional regulation, making music therapy a holistic, family-centred intervention.
11	Uggla et al. 2019 [82]	Sweden	Qualitative study	To investigate the subjective experiences of children undergoing hematopoietic stem cell transplantation, parents, and a music therapist during MT interventions and to identify important components and potential common threads in these interactions.	Three main themes emerged: experiences of competency and recognition of self, interactive affect regulation as change potential, and importance of the therapeutic relationship. Music therapy was a valuable and beneficial experience, offering an important way to cope with and manage time spent in hospital.	The intervention was primarily focused on the children with cancer, although parents and siblings were welcome to attend. The study showed that the entire family is affected, and it would be desirable to develop and evaluate music therapy interventions that encompass the whole family.
12	Zanchi et al. 2024 [74]	Italy	Prospective observational design	To investigate the impact of MT on the psychological well-being of children with cancer.	Network analysis showed different patterns of interactions between parameters during the sessions, highlighting positive emotions, calm settings, the child’s ability to take the initiative, their sense of agency, and the parent’s role in guiding them. Significant differences were recorded at each time point between all variables.	Parents, mainly, were present and played a supportive role in the music therapy sessions. The quality of the parent-child relationship was observed as part of the study, and this relationship became more important as the session continued.
13	Docherty et al. 2013* [83]	USA	Qualitative descriptive	Parental perspectives on helpfulness and meaningfulness of creating therapeutic music videos (TMV) with adolescents and young adults undergoing stem cell transplantation.	MT as a confrontational coping strategy facilitated self-reflection, improved QoL and reduced stress during transplantation, improved communication with the social environment, and enhanced the child’s autonomy and independence. Parents also indirectly benefited from these advantages.	Parents observed and interacted with their AYA. They were able to describe mechanisms through which the intervention was helpful and meaningful for the AYA as well as indirect personal benefits.
14	Haase et al. 2020* [84]	USA	Empirical phenomenology study	AYA perspectives on the creation of TMV undergoing stem cell transplantation.	AYAs reported that TMV interventions helped them overcome challenges through opportunities to connect with family, friends, and carers, identify personal strengths, and share their story with others.	AYAs appreciated being able to decide for themselves whether they want to involve family members in the TMV process, which provides an opportunity to interact and make meaningful connections. Presenting TMV elicited emotional responses from family members.
15	Robb et al. 2014* [12]	USA	RCT	To examine the efficacy of the TMV intervention during the acute phase of hematopoietic stem cell transplant.	100 days after transplant the TMV group reported significantly better social integration and family environment, as well as moderate non-significant effect sizes for spiritual perspective and self-transcendence.	TMV intervention involved families through optional participation in the creative process and the video premiere session. This flexible involvement aimed to strengthen family connections.
16	Robb et al. 2017* [69]	USA	Feasibility and acceptability RCT	Feasibility and acceptability of parent-led active music engagement (AME+P) for emotional distress, mood and traumatic stress symptoms.	AME+P was feasible but not acceptable to parents, children’s emotional distress was lower in AME+P than in Audio Storybook Interventions (ASB), parents were more stressed in AME+P than in ASB.	A parent education/coaching component was added to the AME interventions that had already been tested in previous trials and in which parents were actively involved. Parents were trained to deliver the interventions alone with their child.
17	Robb et al. 2023a* [71]	USA	Multicentre RCT	To evaluate the effect of an AME+P intervention on biomarkers of stress and immune function in children with cancer and their parents.	No results yet; however, the study is expected to improve understanding of the impact of active music on various biomarkers and the understanding of dose-response effects. Implementation challenges described.	Parents are actively involved, both as participants and as supporters of their child’s engagement. Their role is essential to understanding the interrelated stress between parent and child during cancer treatment.
18	Robb et al. 2023b* [72]	USA	Multicentre RCT, mixed methods	To investigate the impact of proximal/distal mediators and moderators of AME+P intervention on child/parent distress, quality of life and family function.	There were no significant mediation or moderation effects. However, AME+P is a promising intervention for parents who screen highly for traumatic stress and whose children are more severely affected by hospitalisation. The intervention was well accepted and feasible.	Parents participated directly in sessions with their children and provided important data on emotional distress, well-being, and family functioning. The intervention helped parents whose children were highly distressed or who were stressed themselves.

Note: *AME+P* Active Music Engagement + Parents, *ASB* Audio Storybook, *AYA* adolescents and young adults, *CG* Control Group, *IG* Intervention Group, *MT* Music Therapy, *MTCD* Music Therapy CD, *PedsQL* Paediatric Quality of Life Inventory, *RCT* Randomised Controlled Trial, *TMV* Therapeutic Music Video, *QoL* Quality of Life.

*These studies form part of a collaborative research programme within the Children’s Oncology Group (COG), the world’s largest paediatric oncology research consortium, and are interconnected in terms of both content and structure.

**Table 3.** Characteristics of included studies.

#	Author and Year	Participants	Diagnosis	Location of interventions	Outcome Measure	Interventions
1	Barry et al., 2010 [76]	11 children aged 6 to 13, 5 in the music therapy group, 6 in the standard care group.	Brain tumours (5), kidney tumours (2), leukaemia (2), bone and soft tissue tumours (2).	Peter MacCallum Cancer Centre Melbourne, Australia.	Demographic information, paediatric interview (including Kidcope), parent questionnaires, radiotherapy planning and treatment staff questionnaire, and the music therapist-researcher’s clinical reflexive journal.	Outpatient setting for radiotherapy. Pre-treatment, treatment and post-treatment. The IG created their own music with a music therapist in the waiting room using interactive computer-based software. Sessions lasted 10–90 minutes. After the session, the music therapist ‘remixed’ the music. The MTCD was listened to for 20–90 minutes during radiotherapy and used at home. CG received routine supportive care.
2	Boyde et al., 2024 [70]	52 children aged 5 to 13 years and one family member.	Cancer diagnosis not specified.	Gemeinschaftskrankenhaus Herdecke, Clinic Centre Dortmund, Vestic Children’s Hospital Datteln, Nordoff/Robbins Music Therapy Centre Witten, Germany.	Music-based APCI. Psychometric questionnaires DEMO, GAS, KINDL and EXIS for children and family members, BAS, SCL-9k and WIRF for family members. Feasibility will be described in qualitative terms.	Inpatient or outpatient setting. The APCI is assessed at the beginning and at the end with children and family members. In between, process-oriented MT sessions, in the IG with the participation of a family member, in the CG without. Questionnaires are collected before, after and at follow-up.
3	Fedhila et al. 2023 [73]	20 children from 2 to 14 years.	Haematological malignancies (3), solid tumours (17).	Bechir Hamza Children’s Hospital Tunis, Tunisia.	PedsQL Module Cancer.	Four active or passive MT sessions a week for about 20 minutes. The child’s respiratory and heart rates were measured before and after each session. Children or parents completed the PedsQL questionnaires before the first and after the last MT.
4	Giordano et al. 2021 [75]	20 children from 2 to 15 and their mothers.	Cancer diagnosis not specified.	University Hospital (Polyclinic) in Bari, Italy.	Preoperative anxiety score m-YPAS scale, Self-assessment questionnaire, medication analysis	In T1 and T3, two pre-operative sessions of interactive MT based on the free improvisation therapy model lasting 15–20 min, in T2 two sessions in which videos of the clinic staff were shown.
5	Heiderscheit 2022 [77]	10 children aged 6 to 13 and one parent.	Leukaemia (6), Neuroblastoma (1), Hurler syndrome (1), Rhabdomyosarcoma (1), Lymphoma (1).	Paediatric medical centre in the Midwest, USA.	Journal forms of reporting to explore self-directed active music interventions and the resulting needs.	Inpatient setting during bone marrow transplant. Participants reported engaging in active music making 3–4 times per week and completed 121 journal forms.
6	Mahoney 2019 [78]	One patient, 8 years old.	Acute myeloid leukaemia.	Children’s Hospital of Philadelphia, USA.	Descriptive case report.	4 years of patient-centred group and individual therapy. Three new approaches have been developed: the inclusion of 1) toys, 2) music technology and 3) the family in MT.
7	O’Callaghan et al. 2010 [7]	26 patients, median age 5.7 years and 28 parents.	Leukaemia, neuronal and non-neuronal solid tumours and lymphomas.	Peter MacCallum Cancer Centre, Monash Children’s Hospital Cancer Centre and the Royal Children’s Hospital, Melbourne, Australia.	Data included transcripts from semi-structured research interviews and observations of children’s music behaviours. Qualitative inter-rater reliability was integrated. Findings were compared with music therapists’ perspectives examined elsewhere.	Inpatient and outpatient interviews during chemotherapy, radiotherapy, surgery or bone marrow transplant. Ten patients played instruments, sang, and/or danced during interviews. The multi-perspective analysis is based on ‘grounded theory’ and ‘mosaic research’.
8	O’Callaghan et al. 2016 [79]	138 participants: 26 children and 28 parents of children with cancer, 12 adolescents and young adults with cancer, 52 adults with cancer, 12 carers, and 8 bereaved.	Cancer diagnosis not specified.	Peter MacCallum Cancer Centre, Monash Children’s Hospital Cancer Centre and the Royal Children’s Hospital, St Vincent’s Hospital, Melbourne, Australia.	The study synthesised the qualitative findings, identifying conceptual themes such as the role of music in supporting emotional well-being, fostering a sense of connection, and helping individuals to cope with the effects of cancer.	The research design of each of the five primary studies was informed by grounded theory. Participants’ statements in each study were analysed through an iterative process of coding, category, and thematic development.
9	Rodriguez-Rodriguez et al. 2023 [80]	27 children aged 6 to 18 and 23 parents.	Acute lymphoblastic leukaemia (8), Acute myeloid leukaemia (4), Ependymoma (4), Ewing’s sarcoma (3), Medulloblastoma (3), Neuroblastoma (2), Rhabdomyosarcoma (1), Non-Hodgkin’s lymphoma (1), Langerhans cell histiocytosis (1).	General University Hospital of Alicante, Spain.	Data analysis using MAXQDA, semi-structured interviews with both children and their parents, children’s self-reported emotions using a feelings thermometer, video analysis of facial expressions and behaviour.	A PAR design was used, with 65 MT sessions (21 online), interviews after each session lasted 10 to 15 minutes and feelings thermometer before and after each session, video analysis.
10	Uggla 2019 [81]	A total of 38 children aged two months to 18 years, parents and 6 participants from staff.	Cancer diagnosis not specified.	Astrid Lindgren Children’s Hospital of Karolinska University Hospital-Huddinge, Stockholm, Sweden.	Exploration of objective endpoints as heart rate, blood pressure and saturation, subjective endpoints of pain, mood and health-related quality of life, qualitative interview with children and parents and focus interviews with members of the staff.	To measure physical variables and analyse differences between a music therapy group and a control group. Assess subjective experiences concerning health and QoL at admission, discharge and 6 months after treatment. To explore the interactions between child, parent and therapist in music therapy sessions, focusing on dynamics and effects. Investigate healthcare staff perceptions on music therapy’s emotional and psychosocial value.
11	Uggla et al. 2019 [82]	6 children aged 1 to 18 years with their families.	Cancer diagnosis not specified.	Karolinska University Hospital-Huddinge, Stockholm, Sweden.	Collaborative research interviews, a qualitative method involving children, their parents, and the music therapists.	Active and receptive, person-centred MT sessions with singing, listening, playing instruments, improvisation, song composition and involvement of family members. Interviews took place 7–13 months after intervention and lasted 45–60 min.
12	Zanchi et al. 2024 [74]	50 children aged 4 to 6 and one family member.	Cancer diagnosis not specified.	Paediatric Oncology and Haematology Unit at Policlinico S. Orsola Hospital in Bologna, Italy.	AQR and m-YPAS to assess crucial parameters like arousal, eye contact, and interaction with parents and physicians.	The interactive-relational approach with therapist and co-therapist is based on the clinical sound-music therapy model. The impact of MT sessions on behaviour, sound/music and interaction was evaluated. Data were collected from T0 (pre-intervention) to T4 (post-intervention).
13	Docherty et al. 2013* [83]	16 parents (30 to 51 years) of AYAs (11 to 24 years).	Cancer diagnosis not specified.	6 paediatric and 3 adult hospitals across the USA.	Semi-structured open-ended interviews of 20 to 60 minutes between 100 and 160 days post-transplantation. The study included the benefits of TMV for AYAs, parents, and feasibility of the intervention during the acute phase of transplantation.	Interviews addressed the six-session TMV creation process during stem cell transplantation.
14	Haase et al. 2020* [84]	14 AYAs aged 13 to 22.	Cancer diagnosis not specified.	Not specified.	Semi-structured interviews lasting 4 to 24 minutes were conducted. The initial question is open-ended to generate data; during the interview, follow-up questions were asked to deepen participants’ reflections.	Inpatient setting during stem cell transplantation. IG group created TMV, standard group had standard treatment, and CG listened to audiobooks.
15	Robb et al. 2014* [12]	113 patients aged 11 to 24 years, 59 in the intervention group, 54 in the control group.	Leukaemia, lymphoma and solid tumours.	Riley Children’s Hospital and Indiana University Hospital, Indianapolis, Children’s Mercy Hospitals and Clinics, Kansas City, Children’s Healthcare of Atlanta/Emory University, Atlanta, Methodist Children’s Hospital and Texas Transplant Institute, San Antonio, St. Louis Children’s Hospital and Barnes-Jewish Hospital, St. Louis, Duke Children’s Hospital, Durham, Helen DeVos Children’s Hospital, Grand Rapids, C.S. Mott Children’s Hospital, Ann Arbor, USA.	RIM, McCorkle Symptom Distress Scale, Mishel Uncertainty in Illness Scale, Jalowiec Coping Scale-revised, Reed Spiritual Perspective Scale, Perceived Social Support (Health Care Providers, Friends, Family), Family Adaptability/Cohesion Scale II, Parent-Adolescent Communication Scale, Herth Hope Index, Reed Self-Transcendence Scale, Haase Resilience in Illness Scale.	6 MT or ASB sessions over three weeks, with data collection at baseline, after the intervention, and 100 days after the transplant. The study was guided by the Resilience in Illness Model (RIM). AME Interventions based on Contextual Support Model of Music Therapy (CSM-MT) by Robb.
16	Robb et al. 2017* [69]	9 children aged 3 to 8 with one parent in AME+P, 7 children aged 3 to 8 with one parent in ASB.	Leukaemia or tumours.	Riley Hospital for Children at Indiana University Health, Indianapolis, USA.	AME Parent Delivery Checklist, Positive Facial Affect and Child Engagement, POMS-SF, IES-R, parent interviews at intervention and 30-day follow-up.	Inpatient setting during chemotherapy. Three 45-minute AME+P sessions using a resource kit, leaflets and active guidance, or 35-minute ASB audiovisual stimulation control group. AME Interventions based on Contextual Support Model of Music Therapy (CSM-MT) by Robb and Kazak’s Paediatric Medical Traumatic Stress Model.
17	Robb et al. 2023a* [71]	228 children aged 3 to 8 and one parent.	Acute lymphoblastic leukaemia, Lymphoma.	Three children’s hospitals in the USA.	Parent questionnaires; cortisol samples from parents and children before and after interventions; blood samples from children after sessions 1 and 4 (standard risk) or 1 and 8 (high risk).	One weekly session of 30 minutes AME+P; one weekly session of 20 minutes ASB. 4 weeks for standard-risk participants and 8 weeks for high-risk participants.
18	Robb et al. 2023b* [72]	125 children aged 3 to 8 and one parent.	Acute lymphoblastic leukaemia, Lymphoma.	Riley Hospital for Children Indiana, Children’s Mercy Hospital Kansas City, Children’s Healthcare of Atlanta, MD Anderson Children’s Cancer Hospital Houston, USA.	CHQ Mental Health Subscale and KINDL questionnaire for children. POMS-SF, IES-R and Index of Well-being for parents. Family functioning was assessed using FACES II. Feasibility and indirect impacts will be described qualitatively.	Three sessions of AME+P or ASB. Pre-, post- and follow-up assessment of proximal mediators (parent-child dyad engagement), distal mediators (family functioning, self-efficacy, independent AME+P use) and moderators (stress from previous hospitalisation, traumatic stress symptoms, child age).

Note: AME+P Active Music Engagement + Parents, APCI Assessment of Parent–child Interaction, ASB Audio Storybook, AQR Assessment of the Quality of Relationships, AYA adolescents and young adults, BAS Burden Assessment Scale, CG Control Group, CHQ Child Health Questionnaire, CSM-MT Contextual Support Model of Music Therapy, DEMO Demographic Data, EXIS Experience in Social Systems, FACES II Family Cohesion Scale, GAS Goal Attainment Scale, IES-R Impact of Events Scale-Revised, IG Intervention Group, Kidcope checklist for cognitive and behavioural coping in children and adolescents, KINDL Children and Young People Quality of Life, m-YPAS Modified Yale Pre-Operative Anxiety Scale, MAXQDA Max Weber Qualitative Data Analysis, MT Music Therapy, MTCD Music Therapy CD, PAR Participatory Action Research, PedsQL Paediatric Quality of Life Inventory, POMS-SF Profile of Mood States-Short Form, RIM Resilience in Illness Model, SCL-9k Symptom Checklist, TMV Therapeutic Music Video, QoL Quality of Life, WIRF Witten Resources Questionnaire.

*These studies form part of a collaborative research programme within the Children’s Oncology Group (COG), the world’s largest paediatric oncology research consortium, and are interconnected in terms of both content and structure.

**Table 4.** Implications and recommendations for future research and clinical practice.

#	Author and Year	Research	Clinical Practice
1	Barry et al., 2010 [76]	Conduct larger studies with objective measures, independent data collection, and control groups; explore long-term effects and parental involvement.	Integrate structured, child-friendly music therapy into standard care to reduce anxiety and improve coping and satisfaction.
2	Boyde et al., 2024 [70]	Use larger, diverse samples and longitudinal designs; compare approaches and assess long-term outcomes of interaction-focused MT.	Embed MT into family-centred oncology care with interprofessional collaboration, supervision, and ongoing training.
3	Fedhila et al. 2023 [73]	Conduct larger, controlled studies to confirm MT’s effects; extend sample size and duration.	Implement MT routinely as a simple, non-invasive method to enhance quality of life and reduce stress.
4	Giordano et al. 2021 [75]	Investigate MT effects during invasive procedures, focusing on sedation, anxiety, and parent – child interaction.	Integrate MT as a non-pharmacological anxiety-reduction method led by trained therapists in paediatric oncology.
5	Heiderscheit 2022 [77]	Evaluate family-directed active music-making in larger controlled trials; include cost-effectiveness analysis.	Implement patient- and family-led MT in hospitals; ensure flexibility, cultural sensitivity, and professional guidance.
6	Mahoney 2019 [78]	Explore flexible, developmentally tailored MT using creative and family-based approaches.	Apply adaptive, relationship-oriented MT supporting self-expression, family inclusion, and meaning-making.
7	O’Callaghan et al. 2010 [7]	Investigate MT across cultures and settings; include children’s voices and therapist effects.	Expand MT and musical support in paediatric oncology; foster comforting sound environments and parental use of music.
8	O’Callaghan et al. 2016 [79]	Study when and how music helps or burdens patients and families; explore home and self-care contexts.	Offer individualized MT, guiding families to use music safely and meaningfully within care routines.
9	Rodriguez-Rodriguez et al. 2023 [80]	Explore healthcare providers’ perspectives and digital MT formats; evaluate broader accessibility.	Encourage MT integration into care routines; allow children to choose music and instruments for better engagement.
10	Uggla 2019 [81]	Investigate MT during and after stem cell transplantation using larger mixed methods designs.	Apply individualized, family-inclusive MT to support communication, resilience, and nonverbal expression.
11	Uggla et al. 2019 [82]	Study timing, dosage, and systemic family effects of MT during transplantation.	Implement family-centred MT promoting safety, belonging, and adaptive coping during treatment.
12	Zanchi et al. 2024 [74]	Conduct multicentre studies on MT effects considering gender, tumour type, and resilience factors.	Integrate MT to strengthen emotional expression, self-efficacy, and family connection; involve parents actively.
13	Docherty et al. 2013* [83]	Examine long-term effects of MT on communication, coping, and resilience in AYA; include varied samples and contexts.	Use MT flexibly to promote structure, autonomy, and family connection; adapt interventions to individual needs.
14	Haase et al. 2020* [84]	Study how songwriting interventions can be tailored to AYA needs; use robust designs to assess relational effects.	Design individualized, flexible MT interventions fostering connection, creativity, and emotional regulation.
15	Robb et al. 2014* [12]	Extend TMV research to broader AYA groups; include long-term and cost-effectiveness analyses.	Use therapeutic music videos to build resilience, hope, and family connection within care teams.
16	Robb et al. 2017* [69]	Explore MT’s impact on parent – child coping and parental stress; use larger, longer trials.	Deliver MT primarily by qualified therapists; create flexible, child-centred sessions supporting family bonding.
17	Robb et al. 2023a* [71]	Examine biological mechanisms and optimal ‘dose’ of MT affecting stress and immune responses.	Integrate MT as an evidence-based tool to reduce distress and support emotional and physiological recovery.
18	Robb et al. 2023b* [72]	Investigate mediators (e.g. parental engagement, stress perception) in AME+P interventions.	Use AME+P early in treatment to support parents under stress; conduct regular stress screenings.

Note: AME+P Active Music Engagement + Parents, AYA adolescents and young adults, MT Music Therapy, TMV Therapeutic Music Video.

*These studies form part of a collaborative research programme within the Children’s Oncology Group (COG), the world’s largest paediatric oncology research consortium, and are interconnected in terms of both content and structure.

To classify the level of family involvement within each study, the distinction between *participating observer* and *observing participant* is applied. According to this principle, the *participating observer* primarily assumes an observational role and only secondarily acts within the intervention, whereas the *observing participant* functions mainly as an active agent while simultaneously maintaining an element of observation [66,67].

### Data extraction integrity

To standardise and ensure the quality of data extraction in this scoping review, we developed a *Rationale for Information Extraction* (Supplementary Material 1). Data were extracted systematically and transparently in accordance with PRISMA-ScR using predefined extraction forms covering the *Summary of included studies* (Table 2), *Characteristics of included studies* (Table 3), and *Implications and Recommendations for Future Research and Clinical Practice* (Table 4). After full review of all included papers, the newly extracted information was cross-checked against the primary author’s extraction; discrepancies were evaluated and the level of agreement was rated on a scale from 50% (inadequate) to 100% (excellent). Two raters independently assessed agreement across all categories, and inter-rater reliability was calculated as percentage agreement [68]. More robust indices (e.g. Cohen’s kappa) were not applied due to the low level of overall observer variance. Agreement between raters ranged from 83% to 100% across studies, categories and tables. Mean extraction integrity for Table 2 was 94% (range: 78%–100%), for Table 3 94% (range: 80%–100%), and for Table 4 also 94% (range: 73%–100%). Overall extraction integrity across all ratings was 94% (range: 79%-100%) (Supplementary Material 2).

## Results

### Overview of included studies

Tables 2 and 3 summarise the included studies and their key characteristics, including country of origin, sample features, and diagnoses.

The analysed studies are conducted across seven countries. The largest proportion originates from the United States, accounting for eight studies (44%), followed by Australia with three studies (17%). Two studies each are conducted in Italy (11%) and Sweden (11%), while individual studies are identified from Germany, Spain, and Tunisia (6% each).

The included studies cover a broad age range, from infants approximately two months old to adolescents and young adults up to 24 years of age. Across the studies, solid tumours represent the largest diagnostic group, accounting for approximately 25%. Acute lymphoblastic leukaemia constitutes around 12%, other forms of leukaemia approximately 9%, and brain tumours about 7%. Ependymomas account for roughly 6%, while the remaining diagnoses – such as acute myeloid leukaemia, neuroblastoma, rhabdomyosarcoma, lymphomas, and several single rare diagnoses – each represent only a small proportion of the total sample.

#### Interpretative synthesis across studies

The included studies reveal a clear geographical imbalance in the current evidence base. Most studies originate from a small number of selected countries, mainly in the Global North and in high-income settings, while low- and middle-income countries are only minimally represented and low-income countries are almost entirely absent. This pattern limits the transferability of the findings to resource-constrained settings and leaves possible cultural differences in family participation largely unexplored.

### Study designs and methodological approaches

Table 2 outlines the methodological designs of the included studies, ranging from randomised controlled trials to qualitative and participatory approaches.

#### Randomised controlled trials (RCTs)

Five studies are conducted as randomised controlled trials [12,69–72].

#### Quasi-experimental, observational, and prospective designs

In addition, quasi-experimental approaches [73], observational designs [74] and prospective studies [75] are identified.

#### Qualitative and participatory research

A considerable proportion of the included studies employ qualitative designs, including interview studies [7,76,82,83,84], journal-based methods [77], and participatory research approaches [80].

#### Synthesis and single-case studies

The dataset also includes synthesis-based and single-case work, such as a meta-ethnographic analysis [79], a single-case study [78], and a doctoral dissertation comprising several sub-studies [81].

#### Multicentre studies

Five of the analysed studies are conducted in multicentre settings [7,70,71,72,79].

#### Interpretative synthesis across studies

Taken together, the included studies suggest that the evidence base is characterised by relatively few randomised controlled trials, several of which originate from the same research team, and a predominance of qualitative, exploratory, and descriptive designs. Overall, this pattern indicates that research in this field is still at an early stage and has so far focused more strongly on experiences, relational processes, and feasibility than on broadly comparable effectiveness data.

### Study objectives

Study objectives across the included literature are summarised in Table 2 and span psychological, physiological, and family – social domains as well as feasibility-related aims.

#### Psychological

The studies investigate anxiety, stress, depressive symptoms, traumatic stress, emotional expression, withdrawal tendencies, resilience, and self-efficacy. These psychological outcomes are examined across qualitative, observational, and experimental designs [7,12,69,72,74,75,76,78,80,81,83,84].

#### Physiological

Several studies focus on somatic stress responses and regulatory processes, including heart and respiratory rate, cortisol and immune parameters, pain perception, and sleep quality [71,73,81].

#### Family – social

The studies examine interaction, communication patterns, relationship quality, and the development of familial coping resources during the illness trajectory. This includes research on dyadic regulation, family meaning-making, and parental well-being [7,69,70,72,77,79,80,82,83].

#### Feasibility

Several studies also assess the feasibility, acceptability, and implementation of music therapy interventions within clinical settings [69,70,72,74,75,77].

#### Interpretative synthesis across studies

Taken together, the included studies suggest that this is still an emerging field, reflected in the frequent focus on feasibility and acceptability. Study objectives are directed mainly at psychological and relational outcomes, while biological mechanisms are explored only in initial ways through basic physiological and biomarker measures. Overall, the evidence base appears more focused on clinically relevant and practicable outcomes than on complex mechanistic investigation.

### Characteristics of interventions

Table 3 details the characteristics of the music therapy interventions, including active, receptive, and mixed formats.

#### Expressive music therapy interventions

Most of the studies include expressive and participatory music therapy interventions. These encompass a wide range of musical activities, such as playing instruments, singing and improvisation [70,74,77,78,80], *Active Music Engagement* (AME) [69,71,72], *Therapeutic Music Video* (TMV) [12,83,84] and *Music Therapy CD* (MTCD) [76]. The variety of active methods makes it clear that musical participation is a central feature of the interventions in most studies.

#### Receptive intervention

The included studies do not report on purely receptive music therapy formats as independent interventions. Receptive elements, such as listening to live or recorded music, only occur in specific situations as supplementary components.

#### Mixed intervention

Several studies describe mixed music therapy approaches that combine active and receptive elements flexibly. These formats enable participants to switch between musical participation and listening depending on their resilience, current state of health or medical conditions [7,73,75,79,81,82]. This flexibility enables individual adaptation to clinical and psychosocial requirements.

#### Interpretative synthesis across studies

Taken together, the included studies suggest that most interventions are expressive and interaction-oriented rather than purely receptive. Musical participation, improvisation, songwriting, and shared music-making appear to be central features across studies, whereas receptive elements are more often incorporated as supportive or complementary components. Overall, the intervention landscape reflects a flexible and relational understanding of music therapy that is adapted to the clinical condition, psychosocial needs, and family context of paediatric oncology care.

### Data collection and analysis methods

Outcome measures and data collection/analysis methods used across studies are summarised in Table 3.

#### Quantitative assessment

Standardised questionnaires on quality of life, such as the *PedsQL* and *KINDL*, are applied in several studies [70,73]. Scales assessing anxiety and stress, including the *m-YPAS* and *POMS-SF*, are used to capture psychological responses [69,75]. Physiological parameters such as heart rate, respiratory rate, blood pressure, oxygen saturation, and cortisol levels are recorded [71,73,81]. In addition, family- and interaction-related scales, including *FACES II*, *EXIS*, and the *APCI*, serve the assessment of family dynamics and relationships [12,70].

#### Qualitative assessment

Qualitative data collection focuses on semi-structured interviews with children, parents, and, in some cases, healthcare professionals, complemented by behavioural observations and analyses of musical interaction [7,80]. Some studies employ *Grounded Theory* methodologies or meta-ethnographic synthesis to identify recurring themes [7,79]. Additional data sources include participant diaries [77], reflective journals kept by music therapists [76], and video analyses of expression and behaviour [80].

#### Mixed methods approach

Mixed-methods studies combine quantitative measures with qualitative techniques such as interviews, diaries, or observations. This allows the documentation of both objective outcomes – such as changes in quality of life, anxiety, or stress – and subjective experiences and process descriptions [69,70,72].

#### Interpretative synthesis across studies

Taken together, the included studies suggest that data collection in this field combines standardised psychological and physiological measures with interviews, observations, diaries, and other qualitative approaches. This pattern indicates an effort to capture both measurable outcomes and subjective, relational, and process-oriented dimensions of music therapy in paediatric oncology.

### Main Results

The main findings reported in the included studies are synthesised in Table 2, covering psychological, physiological, and family/social outcomes as well as feasibility and acceptability.

#### Psychological outcomes

Most studies examine psychological effects of music therapy interventions. Reported outcomes include reductions in anxiety, stress, distress, and depressive symptoms, alongside improvements in mood, coping strategies, well-being, and health-related quality of life [7,12,74,76,80,81].

Several studies also describe how music therapy supports emotional expression, identity formation, and resilience, particularly during demanding treatment phases [12,72,83].

A study shows that the absence of music therapy during the pandemic leads to increased anxiety, stress and strain in children and mothers during invasive oncological procedures [75].

#### Physiological outcomes

Several studies investigate physiological responses to music therapy. Reported parameters include heart rate, respiratory rate, blood pressure, and oxygen saturation. Findings indicate stabilisation or improvement of physiological regulation and reductions in stress-related responses in various clinical contexts [73,81]. Additional studies analyse salivary cortisol and immunological markers to explore whether music therapy influences neuroendocrine or immune functioning [71]. These biomarker-focused approaches provide emerging evidence on potential biological mechanisms of action.

#### Family and social outcomes

Approximately half of the included studies address family and social dimensions. Reported findings relate to parent – child interaction, communication, relationship quality, and perceived family functioning [12,69,70,72,82,83].

Qualitative accounts describe parents’ and siblings’ perspectives on the supportive, relational, and meaning-making aspects of shared music experiences, including enhanced emotional connection, improved communication, and strengthened family cohesion during treatment [70,80,82,83].

#### Feasibility and acceptability

Several studies assess the feasibility and acceptability of music therapy programmes within paediatric oncology settings. Indicators include participation rates, session adherence, parental engagement, and family feedback on the implementation of interactive or parent-directed formats [69,72,77]. Studies employing qualitative, descriptive, or case-based designs offer additional contextualisation of the logistical conditions, delivery characteristics, and clinical integration of music therapy interventions [78,79,81]. Parents and medical staff consistently report high acceptability, perceived usefulness, and meaningful integration into the wider care environment [82,83].

#### Interpretative synthesis across studies

Taken together, the included studies suggest that reported benefits are most consistent in psychological and relational domains. Reductions in anxiety, stress, and distress, as well as improvements in emotional expression, communication, and connectedness, are described more frequently than physiological or broader family-system outcomes. At the same time, generally positive findings on feasibility and acceptability indicate that family-oriented music therapy is considered meaningful and practicable across paediatric oncology contexts, although substantial heterogeneity limits direct comparison between studies.

### Family involvement

Modes and levels of family involvement are summarised in Table 2, ranging from primarily observational roles to active participation and parent-led facilitation. Here, observational involvement refers to family members being present as witnesses, respondents, or reflective informants without directly engaging in the musical activities, whereas active participation refers to direct involvement in shared musical interaction or co-regulation during the session.

#### Participating observers

The role of family members is mainly observational or reflective. Parental involvement is recorded through parent-completed questionnaires [73], interviews about experiences without active participation [75], or observation of sessions followed by feedback interviews [80]. Within TMV interventions, parents often reflect on the perceived meaning and impact of the sessions on their children rather than participating directly [83,84].

#### Observing participants

Other studies describe active participation of family members in the therapeutic process. In projects involving the creation of personalised music CDs, relatives accompanied children throughout the creative process [76]. In interaction-focused sessions, parents take part in music therapy on an equal basis with their children [70]. During bone marrow transplantation, musical activities are undertaken jointly by children, parents, siblings, and grandparents [77]. Qualitative accounts also reports family participation during coping or bereavement processes [78] and describe shared music-making as a means of supporting children and activating parental resources [7,79].

Further studies report opportunities for parents and siblings to be actively included during haematopoietic stem cell transplantation, promoting emotional connection and mutual support [81,82]. In daily oncology settings, parents accompany and support their children during music therapy sessions [74]. Family members are also engaged in creative development and presentation of TMVs [12] or receive structured guidance to deliver music activities independently as part of the AME+P programme [69,71,72].

Overall, the studies report a spectrum of family involvement ranging from primarily observational and reflective roles to active participation and, in some interventions, therapist-guided facilitation roles undertaken by parents.

#### Interpretative synthesis across studies

Taken together, the included studies suggest that family involvement functions not merely as contextual support, but as a relational component of music therapy. Studies describing direct or interaction-focused participation tend to report benefits in communication, emotional connectedness, mutual support, and family cohesion. At the same time, the forms and intensity of family involvement vary considerably across studies, indicating that family participation is shaped by the clinical situation, available resources, and the aims of the respective intervention.

### Implications and recommendations for research and clinical practice

Key implications and recommendations derived from the included studies are synthesised in Table 4 for both future research and clinical practice.

#### Research

Several studies recommend future research with larger, methodologically robust and diverse samples to provide more reliable evidence [70,73,76]. Controlled study, comparison groups, and standardised measurements are proposed to obtain valid data [69,75].

Longitudinal investigations are also mentioned to be necessary for evidence about long-term effects of music therapy and potential biological mechanisms, including neuroendocrine and immunological processes [71,83]. Further recommendations address family-centred and interaction-oriented approaches with particular attention to parental participation and its relationship to child well-being [70,72,82].

Several publications also suggest examining different cultural and clinical contexts, as well as including the different perspectives of children, family members, and healthcare professionals to enhance understanding of contextual factors and intervention conditions [7,74,80].

#### Clinical practice

Recommendations for clinical practice describe music therapy as a structured, child-oriented, and creative intervention integrated within routine paediatric oncology care to support anxiety reduction, stress management, emotional expression, and coping [75,76]. The interventions are suggested to be individually adapted and flexible, based on the needs, preferences, and current condition of each child or adolescent [78,82,84].

Several studies emphasise approaches involving active participation of parents to strengthen shared coping and family resilience [69,70]. Integration of music therapy within interdisciplinary teams, cooperation with nursing and psychosocial services, and regular professional supervision and continuing education for therapists are also described [67,77].

Most of the analysed studies refer to music therapy as part of a holistic, evidence-based care model within the family system.

## Discussion

### Interpretation of the findings

This scoping review indicates that the included literature increasingly describes family involvement in music therapy in paediatric oncology as an important component of psychosocial care (RQ1). Across the included studies, music therapy is most consistently associated with clinical psychological and relational benefits. In this sense, family involvement appears not merely as contextual support, but as a relational resource that may strengthen shared coping and family resilience.

The varying forms of family involvement identified across studies (RQ2) further suggest that family participation is a flexible process. The spectrum ranges from primarily observational and reflective roles to direct participation in shared music-making and, in some cases, therapist-guided parent-led facilitation. While this variety complicates direct comparison, it also reflects the need to adapt interventions to the child’s condition, the family’s emotional resources, and the practical realities of treatment.

At the same time, the evidence base is characterised by substantial methodological, conceptual, and contextual heterogeneity. This should not be understood solely as a methodological weakness, but also as a reflection of the realities of paediatric oncology care, where medical instability, emotional strain, and organisational constraints often make highly standardised research difficult. In this context, a *Real-World Approach* perspective is particularly relevant, as controlled experimental conditions are often not feasible in clinical settings and research in practice environments involves complex and uncontrollable contextual factors that require methodological adaptation [76,85,86]. Importantly, this does not mean that only larger and highly standardised studies are methodologically valuable. Rather, smaller, qualitative, exploratory, and mixed-methods studies may be more compatible with clinical reality and are often better suited to capturing the lived, relational, and process-oriented dimensions of family-centred music therapy in paediatric oncology [76,85,86].

From a global child health perspective, the review also points to an important imbalance in the geography of evidence. The included studies are concentrated primarily in high-income settings, with only very limited representation beyond these contexts. As a result, the transferability of findings to low-resource settings remains uncertain, and the current evidence base reflects broader inequities in psychosocial oncology care and in the production of supportive care research [13].

### Clinical implications

The findings support the clinical relevance of family-oriented music therapy as part of interdisciplinary paediatric oncology care. Involving family members was most often associated with enhanced communication, emotional regulation, mutual support, and shared coping. Clinically, this suggests that music therapy should not be understood only as an individual supportive intervention for the child, but also as a flexible family-oriented resource that can be adapted to different levels of participation.

This perspective is also relevant across different health-system settings. In contexts where specialist psychosocial services are limited or unevenly available, family-integrated and parent-guided music therapy approaches may offer a meaningful way to extend psychosocial support beyond individual bedside interventions. At the same time, such approaches should not be viewed as substitutes for professional psychosocial care. Their implementation requires training, supervision, contextual adaptation, and integration into interdisciplinary care structures. From a broader systems perspective, the findings therefore support stronger structural anchoring of music therapy within paediatric oncology services in line with patient- and family-centred care principles [14] and underscore the need for wider efforts to improve equitable access to psychosocial care for children with cancer and their families, particularly across resource-diverse settings [13].

### Recommendations for future research

The findings also yield important implications for future research (RQ3). Future studies would benefit from greater conceptual clarity and stronger methodological comparability. Clearer definitions of family involvement are needed, including distinctions between observational presence, shared participation, and therapist-guided parent- or family-delivered components. Intervention characteristics should also be reported more systematically, including therapeutic orientation, intensity, duration, delivery format, and the professional qualifications of those providing the intervention [16].

At the same time, the field should not be guided exclusively by the expectation of large, highly standardised trials. While randomised controlled trials remain important for testing effectiveness and informing future guideline development, they are often difficult to implement in paediatric oncology settings. Future research should therefore combine efforts toward greater methodological comparability with study designs that remain feasible in practice. This includes pragmatic, longitudinal, and multicentre approaches, but also smaller qualitative, exploratory, and mixed-methods studies that can generate clinically relevant and practice-near knowledge under real-world care conditions [76,85,86].

Finally, more research is needed in underrepresented and resource-constrained settings, including studies that examine whether and how family participation varies across cultural and health-system contexts, including low-income countries as well. This is important for understanding how family-oriented music therapy can be adapted across different care environments and for clarifying its relevance for equitable psychosocial oncology care worldwide [13].

## Limitations

Several limitations must be considered when interpreting these findings. The included studies exhibit substantial methodological and conceptual heterogeneity. Variations in study design, sample size, intervention type, outcome measures and definitions of family involvement hinder systematic comparability and precluded a quantitative synthesis. Differences in the theoretical orientation, intensity, duration, and professional implementation of music therapy interventions further limit direct comparison across studies. In addition, intervention characteristics were not always described with the same level of detail across studies. Differences in the reporting of therapeutic orientation, intensity, duration, delivery format, and therapist qualifications limited comparability and made it difficult to determine which intervention components may have been most relevant to the reported outcomes.

In line with the aims of a scoping review, no formal appraisal was conducted, preventing assessment of the methodological robustness of individual studies. Thus, given the conceptual, methodological and contextual diversity across studies, this review can provide only a descriptive overview of the research landscape on family involvement in paediatric oncology.

The literature search was restricted to the period from 2010 to 2025 and to publications in German and English. In addition, all searches were conducted in the Abstract field using free-text terms only, which may have reduced sensitivity for potentially relevant studies. An extension to earlier publication years and additional languages would be desirable in future work. Responses to requests for grey literature were limited. Together, these factors may have contributed to the omission of relevant publications.

The screening from initial hits to full-text inclusion was carried out by a single reviewer, which may have increased the risk of selection bias and reduced the reliability of study selection, particularly in borderline cases. However, the review was guided by predefined eligibility criteria, exclusion decisions were transparently documented, and the extracted data were independently verified and rated by multiple raters, which supported the credibility of the resulting synthesis.

## Conclusion

This scoping review suggests that family-centred music therapy may contribute to psychosocial support in paediatric oncology. Shared musical experiences may support emotional stability, facilitate communication, and strengthen dyadic and familial coping processes. At the same time, the available evidence remains heterogeneous and primarily descriptive, limiting conclusions about effectiveness. Research in this area must therefore operate within the realities of clinical practice, where emotional, medical, and organisational constraints are common. In this context, the *Real-World Approach* provides a useful framework for understanding mechanisms that are often less visible in standardised designs. Clinically, the structural integration of family-oriented music therapy into interdisciplinary care pathways could support sustained access in the everyday lives of affected families.

## Supplementary Material

PRISMA ScR Checklist.docx

Supplemental Material.docx

## Data Availability

All relevant data can be obtained by the first author.

## Abbreviations

AME: Active Music Engagement
DGPs: German Psychological Society
DGSF: German Association for Systemic Therapy, Counselling, and Family Therapy
DKPM: German College of Psychosomatic Medicine
GPOH: Society for Paediatric Oncology and Haematology
IGIM: Interprofessional Graduate School of Integrative Medicine and Health Sciences
IQWiG: Institute for Quality and Efficiency in Health Care
MT: Music Therapy
OEGPS: Austrian Psychological Society
OSF: Open Science Framework
PRISMA-ScR: Preferred Reporting Items for Systematic reviews and Meta-Analyses – Scoping Reviews
PSAPOH: Psychosocial Working Group for Paediatric Oncology and Haematology
PSYNDEX: The German Database for Psychology and Behavioural Sciences
SG: Systemic Society
TMV: Therapeutic Music Video
