# Supplementary material for: Mapping family involvement in music therapy for children and adolescents with cancer: a scoping review
Source: Glob Health Action. 2026 Jul 9;19(1):2674493. doi: 10.1080/16549716.2026.2674493 (PMC13353383; doi:10.1080/16549716.2026.2674493)
Supplement: Supplemental Material.docx [file ZGHA_A_2674493_SM9902.docx]

**Supplemental Material 1:** *Rationale for Information Extraction*

The information extraction aims to systematically and transparently collect all relevant information from the included studies to address the research questions of this scoping review. In accordance with the PRISMA-ScR guidelines, the following extraction forms are used:

**Table 2:** *Summary of included studies*

| # | Author and Year | Country | Study Design | Objectives | Main Results | Family Involvement |
| --- | --- | --- | --- | --- | --- | --- |

**Table 3:**  *Characteristics of included studies*

| # | Author and Year | Participants | Diagnosis | Location of interventions | Outcome Measure | Interventions |
| --- | --- | --- | --- | --- | --- | --- |

**Table 4:** *Implications and Recommendations for Future Research and Clinical Practice*

| # | Author and Year | Research | Clinical Praxis |
| --- | --- | --- | --- |

Each paper included must be reviewed in full and the information extracted must be organised according to these predefined categories. After completion, the newly gathered information must be compared with the information extracted by the main author to verify accuracy and completeness.

Discrepancies between the new extraction and the main author’s version will be critically assessed. The overall content consistency of the main author’s table must be rated according to the following scale:

| **Agreement Level** | **Rating** | **Description** |
| --- | --- | --- |
| 100% | Excellent | Complete agreement |
| 90% | Very good | Minimal discrepancies |
| 80% | Good | Minor deviations, overall accurate |
| 70% | Satisfactory | Noticeable but acceptable differences |
| 60% | Adequate | Moderate inconsistencies |
| 50% | Inadequate | Major deviations, poor agreement |

Two reviewers should conduct this process independently, resolving disagreements by consensus to ensure methodological reliability and reduce bias. If consensus cannot be reached, the auditor makes the final decision.

**Supplemental Material 2:** *Rating Data*
